# Flour the Grand Panacea for Burns and Scalds

**Published:** 1830-01-01

**Authors:** 


### FLOUR THE GREAT PANACEA FOR BURNS AND SCALDS. By Dr. WARD of Manchester.

Of all enthusiasts, scarcely excepting the religious, surely none are so mad as professional visionaries. Once a week or so we get a certain cure for tetanus or hydrophobia, which the discoverers are not content with devoutly crediting themselves, but they are mightily indignant if any one should evince the slightest hesitation in following their example. For our own parts we are astonished, after all the uniformly successful modes of treatment which are daily issuing from the press for all the obstinate or incurable diseases, that any whatever should remain to be treated. What a happy world it will be by and bye, when nosologies cease to exist; when cancer, scrofula, organic diseases are forgotten; and maladies are put out, as coats are cut in Laputa, on certain and mathematical principles. We are happy to inform our readers that burns and scalds will no longer afford any trouble to themselves or inconvenience to their patients, at least in such civilized countries as manufacture flour and contain kitchen dredgers. The directions for applying the panacea are extracted from a pamphlet published by Dr. Ward; the italics and capitals are all the author's own.

"Remove the clothes from the injured parts as soon as possible, (care being taken to avoid bursting the blisters) then take a common KITCHEN DREDGER, and sprinkle the parts affected with flour, either till the pain subsides, or so much flour is applied as to form a defence or covering from a quarter to half an inch in thickness. *If the holes in the lid of the dredger be too small, or not so numerous as to allow the flour to escape freely, a table spoon, or the fingers, may then be used to sprinkle the flour equally and plentifully upon the burnt or scalded parts.* If the pain be removed by the flour, *(which has hitherto been the effect in every instance,)* the patient may then sleep, or take some mild nourishment, *and as long as the pain is easy nothing more must be done.* When it returns, more flour must be applied to the painful parts, *without disturbing those that are easy;* and this method must be continued as long as is necessary. In slight cases a few days will suffice to effect a cure. In serious and alarming ones, it will often be necessary to continue applying the flour a fortnight or three weeks, or probably longer.

"If the pain do not soon yield after applying a coating of flour of a proper thickness, (see above) the dredging or sprinkling must be continued, without regard to the quantity of flour used, either till ease be obtained, or the quantity be such as, if increased, would be inconvenient from its weight; then wait a while; and at the second and succeeding dredgings or sprinklings, the uppermost *loose* portions of flour, (if any) may be removed before more is put on. A piece of old linen must then be laid over the flour, and such bed-clothes or other coverings as are necessary to keep the patient warm, *but not too hot.*—If the head or face be burnt or scalded, a silk handkerchief forms a proper covering to keep the flour in its place.

"*N. B. In accidents of this kind happening to children, (young infants more particularly,) the use of flour has been found to produce ease and sleep almost immediately, after other means had been tried in vain.*"

That flour thus applied may be very useful in superficial burns we can very well believe, indeed we have used it ourselves many times. The powdered calamine is constantly employed for the same purpose and on the same principle, and although the "kitchen dredger" may not be in requisition, we venture to say that very few "dressers" will be found in our hospitals who are without an apparatus for *dusting* calamine or some other absorbent powder on the burnt or blistered surfaces. A very common and very useful contrivance is a large pill or ointment box, covered with lawn or cambric and filled with the powder in question. Dr. Ward, however, brings forward the flour as a specific, or at least a remedy of universal application. We firmly believe that when the medical millennium, already so nigh, is arrived, when the profession consists of peaceable individuals, and when churchwarden Wakley is allowed on all hands to be a respectable sort of man; when, we repeat, the foregoing desirable events shall come to pass, flour and the kitchen dredger will be the only remedies in use for burns and scalds. Till then, however, we fear that the merits of these matters must not be viewed in so exclusive a light.

---

XXVI.

